# Assessment of the risk of death of *Clarias gariepinus* and *Oreochromis niloticus* pulse-exposed to selected agricultural pesticides

**DOI:** 10.1038/s41598-021-94262-w

**Published:** 2021-07-19

**Authors:** Kingsley Chukwuemeka Kanu, Adebayo Akeem Otitoloju, Nnamdi Henry Amaeze

**Affiliations:** 1 Department of Environmental Management and Toxicology, Michael Okpara University of Agriculture, Umudike, Nigeria; 2 Environmental Toxicology and Pollution Unit, Department of Zoology, University of Lagos, Akoka-Yaba, Nigeria

**Keywords:** Experimental organisms, Biological techniques, Environmental impact

## Abstract

Aquatic organisms are often exposed briefly to high pesticide concentration. Survival time model was used to study risk of death in *C. gariepinus* and *O. niloticus* fingerlings exposed to 24 mg/L atrazine, 42 mg/l mancozeb, 1 mg/L chlorpyrifos and 0.75 µg/L lambda cyhalothrin for 15, 30, 45 and 60 minutes and continuously for 96 hours. Mortality, time-to-death, weight, length, and condition factor of the fingerlings were recorded. Results obtained showed tilapia was more susceptible than catfish to continuous exposure but not pulse exposure. The survival probability of both species was similar when exposed for 15, 30 and 45 minutes (*p* > 0.05) but differed after 60 minutes (*p* < 0.05). Risk of death of catfish exposed briefly to atrazine, mancozeb and chlorpyrifos for 60 minutes was similar to 96 hours continuous exposure, same for tilapia exposed to 1 mg/L chlorpyrifos (*p* > 0.05). Survival probability of tilapia exposed to chlorpyrifos for 15, 30, 45 and 60 minutes was similar (*p* > 0.05) and was not influenced by pulse length. Pesticide hazard and risk of death decreased as fish size (weight, length, and condition factor) increased. Pulse toxicity assessment using survival models could make pesticides exposure assessment more realistic by studying factors that can influence the toxicity of pesticides.

## Introduction

Pesticides are major environmental concern due to their hazard to non-target aquatic organism. Among the different classes of pesticides, insecticides are generally the most toxic while fungicides and herbicides are considered second and third respectively^1,2^. Atrazine, mancozeb, chlorpyrifos and lambda cyhalothrin are commonly used pesticides in Nigeria and globally^3,4^ . Atrazine is a selective and systemic herbicide used as pre and post emergence herbicide^5^. Its half-life in surface water is greater than 100 days at 20 °C^6^ . Atrazine concentration in surface water varies from ppb to ppm with 0.48 mg/L reported in the runoff from Ohio River watershed shortly after application^7^. Mancozeb is a fungicide used to control fungal pathogens affecting several crops^8^. Its half-life in water is less than 2 days in which it breaks down to ethylene bis isothiocyanate sulfide (EBIS) and into ethylene bis isothiocyanate (EBI) by the action of UV light. Other metabolites are ethylene thiourea (ETU), and ethylene urea (EU)^9^. ETU concentration of 5.9–13.8 μg/L was reported in drainage and runnel water close to banana plantation in southeastern Mexico^10^. Chlorpyrifos (O,O-diethyl O-[3,5,6-trichloro-2-pyridyl] phosphorothioate) is a broad-spectrum, organophosphate (OP) insecticide, acaricide and nematicide. The solubility of chlorpyrifos may range from 0.39 to 1.4 mg/L at temperature range of 19.5 to 25°C^11^. In water, chlorpyrifos adsorb to suspended solids and sediment. Chlorpyrifos residue of 0.67 mg/L was reported^12^ in water samples from river Benue, in Adamawa State, Nigeria. Lambda-cyhalothrin (alpha-cyano-3-phenoxybenzyl (Z)-(1RS)—cis 3-(2-chloro-3,3,3-trifluoro propenyl)-2,2-dimethyl cyclo propane carboxylate) is a pyrethroid insecticide and its solubility is 5 × 10^−3^ mg/L and 4 × 10^−3^ mg/L in purified and buffered water respectively^13^. Lambda-cyhalothrin residue of 0.11–0.14 μg/L was detected in water samples from agricultural watersheds in Stanislaus County, California^14^.

The pathway of pesticides into aquatic ecosystem include spray drift, run-off, dry and wet deposition as well as effluent discharge^15^. Exposure of aquatic organisms to pesticides often occurs as pulse, repeated pulse or sequential exposure to high or fluctuating concentrations^16–18^. Pulse exposure may involves one isolated brief exposure^19^ which may last from a couple of hours to a few days depending on the properties of the pesticide and the receiving water bodies^20^. Aquatic organisms may be exposed during the brief period to high pesticide concentration capable of causing death or harm to early life stages^21^. Standard testing procedures are at variance with environmentally relevant pesticide exposure scenarios in the aquatic environment. Standard toxicity tests usually involve maintaining constant exposure conditions to pesticides which limits their relevance to real situations. It does not evaluate survival chances of non-target organisms briefly exposed to high pesticide concentrations often encountered in real life scenarios^19^. Also routine procedures often ignore the role of time in toxicity. Similarly, pesticide exposure assessment use constant exposure toxicity test set-ups to estimate the hazard of the pesticide even though aquatic organisms may be exposed to high or fluctuating pesticides concentrations in pulses of varying length^20^. Pulse exposure testing differs from standardized testing, as the exposure are not constant and include the observation of post-exposure effects after the pulse exposure^22^.

Despite increasing studies on pulse and repeated pulse exposure of aquatic organisms to pesticides or other toxicants spanning over a decade^20,22–25^, little is known on the responses of catfish and Nile tilapia pulse exposed to pesticides and how species difference, pulse length, and size may influence pulse toxicity of pesticides. Most pesticide toxicity studies involving both species were performed using continuous exposure setups. Utilizing pulse exposure during exposure assessment may make the outcome of an ecological risk assessment more realistic^26^. This could improve the ecological risk assessment of pesticides. It could be used to rank the relative susceptibility of aquatic organism to pesticide pulse exposure and enhance the selection of test organisms to provide broad level protection to other non-target organisms within the ecosystem.

Survival analysis is a relevant technique in eco-toxicological studies. Previous authors have shown how time-to-death analysis can be applied in ecotoxicology to study factors that influence the toxicity of toxicants in exposed aquatic organisms^27–29^. But there are very limited research that have applied this technique to study pulsed toxicity and factors that may influence pulsed toxicity in exposed organisms. By noting time-to-death, post exposure observation period can be studied with more statistical power provided by survival analysis. Thus, this study applied survival analysis to provide insights on the response of fish briefly exposed to selected agricultural pesticide. The prognostic factors thought to be related to survival are species difference, pulse length, fish weight, length, and condition factor. Interest lies in elucidating the influence of these explanatory variables on the survival probability of fish. Commercially available pesticide products containing active ingredients of three pesticides classes were used in this study.

## Materials and method

### Experimental design

Survival of fingerlings exposed continuously and briefly to selected agricultural pesticides; atrazine (herbicide), mancozeb (fungicide), chlorpyrifos (organophosphate insecticide), and lambda cyhalothrin (pyrethroid insecticide) was investigated by noting time to death during and after exposure respectively. Survival analysis was used to estimate the influence of pulse length, weight, length, and condition factor on the survival of the fingerlings pulsed-exposed to pesticides. This study was carried out in compliance with the ARRIVE guidelines.

### Test organisms

Catfish *(Clarias gariepinus)* and Nile tilapia (*Oreochromis niloticus*) fingerlings were selected because they are abundant in natural waters and commonly cultivated in fish farms in Nigeria. This facilitates their use in toxicity tests. The fishes were purchased from a fish farm and transported to the laboratory in the morning (7–8 am). They were acclimated to laboratory condition (ambient temperature 27 °C ± 0.5; relative humidity 72 ± 5%; Light: Darkness, 12:12 h) for seven days in plastic aquarium containing borehole water. The water was renewed daily to eliminate excess feed and metabolic byproducts, while the fishes were fed with commercially available fish feed Coppens twice daily ad libitum. They were not fed during the experiment.

### Test pesticides

The formulated pesticides used in the study are commonly used by farmers in Nigeria^3^. They include; Atraz 50FW, a herbicide containing 500 g/L atrazine active ingredient, Z-force, a fungicide containing 80% mancozeb active ingredients, Attacke, a pyrethroid insecticide containing 2.5% lambda cyhalothrin active ingredient and Chloview an organophosphate insecticide containing 40% chlorpyrifos active ingredient.

### Pesticide stock solution

Atrazine, chlorpyrifos and lambda cyhalothrin stock solutions were prepared by making up one milliliter (1 ml) of the pesticides to one litre (1 L), while mancozeb stock solution was prepared by mixing one gram (1 g) with one litre (1L) of distilled water. Stock solutions were subsequently diluted to the appropriate test concentrations used in the study.

### Pulse toxicity test

All toxicity tests were performed following established acute toxicity testing procedure^30^, except in the manner of exposure. Ten *C. gariepinus* and *O. niloticus* fingerlings were exposed in separate duplicate experiments briefly and continuously to pesticide solutions containing 24 mg/L atrazine, 42 mg/l mancozeb, 1 mg/L chlorpyrifos and 0.75 µg/L lambda cyhalothrin nominal concentrations. These concentrations were selected because they were the maximal concentration used in a previous acute toxicity study of the pesticides using *C. gariepinus* which caused maximal effect i.e. 100% mortality (0% survival) for all the pesticides except atrazine which was 85% mortality (15% survival) after continuous exposure for 96 hours.

Different sets of fingerlings were exposed briefly for 15, 30, 45 and 60 minutes with slight modification^31^ and continuously for 96 hours similar to standard toxicity test. After the brief exposure, the exposed fishes were removed, rinsed with clean water, and transferred to plastic aquaria containing clean water and observed for 96 hours^31^. Water was renewed daily. The 96-hours post-exposure observation period allowed the comparison of pulsed exposure toxicity with standard 96-hours toxicity. Mortality and time-to death were checked and recorded every hour for both the pulsed and continuously exposed fishes. Mortality was determined by lack of movement and response to gentle prodding. Weight and length of dead fish were recorded and fingerlings still alive after 96-hours post observation period were right censored while their length and weight were also measured. Fingerlings condition factor (CF) was calculated using the Eq. ^32^:

$$CF=\frac{W}{{L}^{3 }} \times 100$$

W is fish wet weight (g), L is fish total length (cm).

### Statistical analysis

Student T-test was used to test the equality of the weight, length and condition factor between Nile tilapia and catfish exposed to the pesticides. Pearson's correlation was used to measure the strength of association between weight, length and condition factor. Student T-test and Pearson's correlation was performed using SPSS version 22. Survival probability- Survival time response curve was plotted using Statistica version 10. The equality of survival distribution of fish exposed continuous and briefly for 15, 30, 45 and 60 minutes to each pesticides was tested using the log rank test. Cox proportional hazard model was used to model the influence of species, pulse length, weight, fish length, and condition factor on the survival of fishes exposed to pesticides.The SAS procedure LIFETEST and PHREG was used to perform log rank test and fit the Cox PH model respectively. Continuous and pulse exposure were modelled as categorical variables in the LIFETEST procedure, while fish weight, length and condition factor were fitted as continuous variables in the PHEG procedure using SAS version 9.13.

### Ethics approval and consent to participate

Approval was obtained from the Ethical Committee at the College of Medicine, University of Lagos to use the fish species for pesticide toxicity studies (CMUL/ACUREC/10/20/827). All the fish were handled humanely in compliance with Directive 2010/63/EU on the protection of animals used for scientific purposes and the International Council for Laboratory Animal Science (ICLAS) ethical guidelines.

## Results

### Descriptive and correlation analysis of covariates

Table 1 shows the mean weight, length and condition factor of catfish and Nile tilapia fingerlings used in the study. The weight and length of catfish exposed to the pesticides were significantly different (*p* < 0.05) from Nile tilapia except length of fish exposed to mancozeb for 15, 30 and 60 minutes (*p* > 0.05) and weight of fish exposed continuously for 30, 45 and 60 minutes to chlorpyrifos (*p* > 0.05) as well as fish exposed for 60 minutes to lambda cyhalothrin.

**Table 1:** Mean values of weight, length and condition factor of fish species.

Exposure duration	Parameter	Fish species	Pesticides			
			Atrazine	Mancozeb	Chlorpyrifos	Lambda cyhalothrin
Continuous	Weight (g)	Catfish	0.55 ± 0.02	0.5 ± 0.02	0.49 ± 0.02*	0.54 ± 0.02
		Tilapia	0.23 ± 0.01	0.36 ± 0.05	0.37 ± 0.06*	0.17 ± 0.01
	Length (cm)	Catfish	4.25 ± 0.04	3.85 ± 0.04	3.66 ± 0.05	4.13 ± 0.04
		Tilapia	2.11 ± 0.05	2.64 ± 0.14	2.51 ± 0.16	1.89 ± 0.04
	Condition factor	Catfish	0.72 ± 0.04	0.88 ± 0.05	1 ± 0.06	0.78 ± 0.04
		Tilapia	2.46 ± 0.1	1.85 ± 0.1	2.25 ± 0.11	2.51 ± 0.19
15 Minutes	Weight (g)	Catfish	0.55 ± 0.03	0.47 ± 0.03	0.47 ± 0.02	1.01 ± 0.08
		Tilapia	0.4 ± 0.02	0.95 ± 0.13	0.83 ± 0.17	0.63 ± 0.07
	Length (cm)	Catfish	3.87 ± 0.07	4.05 ± 0.09*	4.08 ± 0.07	5.11 ± 0.16
		Tilapia	3 ± 0.08	3.71 ± 0.21*	3.09 ± 0.29	3.2 ± 0.2
	Condition factor	Catfish	0.96 ± 0.05	0.73 ± 0.05	0.7 ± 0.05	0.76 ± 0.05
		Tilapia	1.55 ± 0.1	1.8 ± 0.12	2.41 ± 0.15	2.1 ± 0.23
30 Minutes	Weight (g)	Catfish	0.46 ± 0.02	0.47 ± 0.02	0.44 ± 0.03*	1 ± 0.05
		Tilapia	0.29 ± 0.02	1.03 ± 0.14	0.41 ± 0.07*	0.57 ± 0.07
	Length (cm)	Catfish	3.84 ± 0.09	3.88 ± 0.08*	3.8 ± 0.1	5.03 ± 0.12
		Tilapia	2.31 ± 0.07	4.02 ± 0.25*	2.62 ± 0.22	3.05 ± 0.21
	Condition factor	Catfish	0.84 ± 0.05	0.82 ± 0.05	0.8 ± 0.03	0.83 ± 0.06
		Tilapia	2.31 ± 0.09	1.55 ± 0.12	2.34 ± 0.19	2.18 ± 0.2
45 Minutes	Weight (g)	Catfish	0.43 ± 0.02	0.48 ± 0.03	0.42 ± 0.02*	0.94 ± 0.08
		Tilapia	0.34 ± 0.02	0.69 ± 0.06	0.55 ± 0.17*	0.55 ± 0.07
	Length (cm)	Catfish	3.56 ± 0.06	3.93 ± 0.11	3.75 ± 0.08	5.11 ± 0.18
		Tilapia	2.72 ± 0.07	3.46 ± 0.11	2.77 ± 0.28	3.12 ± 0.19
	Condition factor	Catfish	0.99 ± 0.07	0.83 ± 0.06	0.82 ± 0.05	0.71 ± 0.05
		Tilapia	1.71 ± 0.08	1.63 ± 0.07	1.85 ± 0.17	1.9 ± 0.19
60 Minutes	Weight (g)	Catfish	0.48 ± 0.04	0.47 ± 0.02	0.41 ± 0.02*	0.56 ± 0.03*
		Tilapia	0.34 ± 0.02	0.83 ± 0.06	0.33 ± 0.08*	0.53 ± 0.07*
	Length (cm)	Catfish	3.79 ± 0.09	3.79 ± 0.08*	3.79 ± 0.14	4.86 ± 0.11
		Tilapia	2.68 ± 0.06	3.69 ± 0.12*	2.42 ± 0.22	2.9 ± 0.22
	Condition factor	Catfish	0.89 ± 0.05	0.87 ± 0.05	0.81 ± 0.07	0.5 ± 0.03
		Tilapia	1.76 ± 0.07	1.63 ± 0.05	1.97 ± 0.15	2.27 ± 0.2

Values indicate mean ± standard error, N = 20. Parameters of catfish was significantly different from Nile tilapia (*p* < 0.05), except those marked with asterisk (*) (*p* > 0.05).

Table 2 shows the strength of the relationship between the intrinsic predictor variables. The weight and length of fish exposed to the pesticides was strongly positively correlated. Weight and condition factor of catfish exposed to atrazine and lambda cyhalothrin were moderately positively correlated, while those exposed to mancozeb had low correlation. There was no correlation between weight and condition factor of catfish exposed to chlorpyrifos. Weight and condition factor of Nile tilapia exposed to atrazine and lambda cyhalothrin had strong negative correlation, while the negative correlations were moderate in tilapia exposed to mancozeb and chlorpyrifos. The length and condition of fishes exposed to the pesticides was strongly negatively correlated.

**Table 2:** Correlation between covariates.

Pearson correlation coefficients			Weight	Length	Condition factor
Atrazine	Catfish	Weight	1	.517**	.371**
		Length	.517**	1	− .565**
		Condition factor	.371**	− .565**	1
	Tilapia	Weight	1	.906**	− .649**
		Length	.906**	1	− .892**
		Condition factor	− .649**	− .892**	1
Mancozeb	Catfish	Weight	1	.488**	.256*
		Length	.488**	1	− .689**
		Condition factor	.256*	− .689**	1
	Tilapia	Weight	1	.902**	− .377**
		Length	.902**	1	− .690**
		Condition factor	− .377**	− .690**	1
Chlorpyrifos	Catfish	Weight	1	.565**	.161
		Length	.565**	1	− .686**
		Condition factor	.161	− .686**	1
	Tilapia	Weight	1	.927**	− .401**
		Length	.927**	1	− .615**
		Condition factor	− .401**	− .615**	1
Lambda cyhalothrin	Catfish	Weight	1	.698**	.329**
		Length	.698**	1	− .413**
		Condition factor	.329**	− .413**	1
	Tilapia	Weight	1	.960**	− .630**
		Length	.960**	1	− .785**
		Condition factor	− .630**	− .785**	1

*Correlation is significant at the 0.05 level (2-tailed).

**Correlation is significant at the 0.01 level (2-tailed).

### Survival analysis

#### Survival probability- survival time response

**Figure 1:**
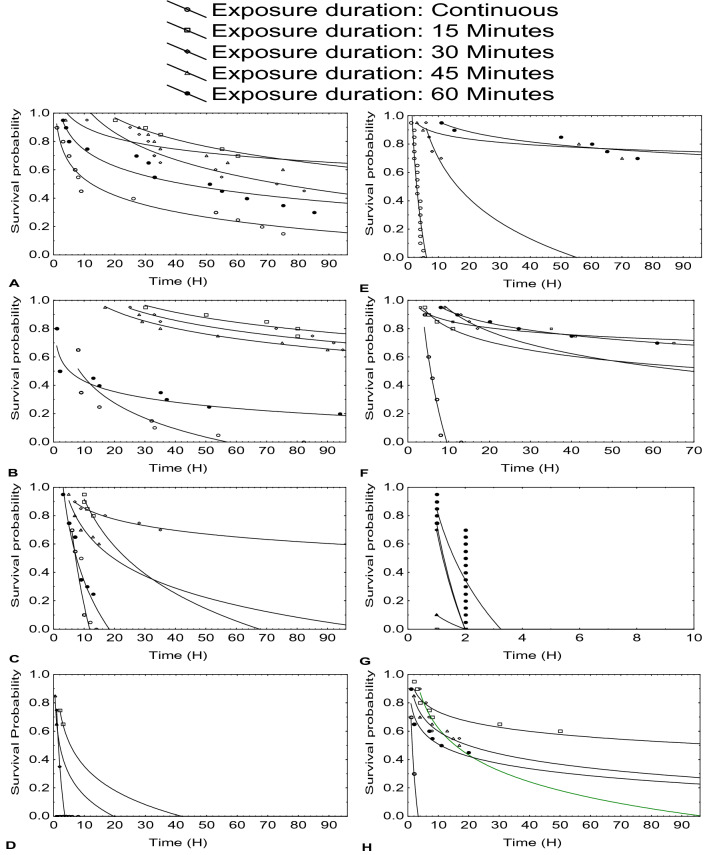
Survival probability-Time curve of catfish exposed to atrazine (**A**), mancozeb (**B**), chlorpyrifos (**C**), lambda cyhalothrin (**D**), and Nile tilapia exposed to atrazine (**E**), mancozeb (**F**), chlorpyrifos (**G**) and lambda cyhalothrin (**H**). Unit of pesticide concentration is mg/L, except lambda cyhalothrin which is µg/L.

Figure 1 shows survival time and probability of survival decreased as pulse length increased. Probability of survival decreased as exposure time increased. Both species were more sensitive to insecticides (Fig. 1c,d,g,h) than herbicides (Fig. 1a,e) and fungicides (Fig. 1b,f). Survival probability was significantly higher for Nile tilapia than catfish following pulse exposure to atrazine, mancozeb and lambda cyhalothrin, however catfish exposed to chlorpyrifos had a lower risk to death than Nile tilapia.

The log rank test indicate that the risk of death from continuous exposure was higher than pulse exposure for both catfish and Nile tilapia fingerlings (*p* < 0.01). Be that as it may, the risk of death of catfish continuously exposed to atrazine, mancozeb and chlorpyrifos were  similar (*p* > 0.05) to those pulse exposed to the pesticides for 60 minutes. Similarly, the risk of death of tilapia continuously exposed to chlorpyrifos was also similar (*p* > 0.05) to 60 minutes pulse exposure. This suggests pulse exposure of 60 minutes was as hazardous as continuous exposure for 96 hours. On the other hand, risk of death of pulse exposure for 15, 30, 45 minutes differed significantly from 60 minutes pulse exposure for catfish exposed to atrazine, mancozeb, and lambda cyhalothrin; and both species exposed to chlorpyrifos (*p* < 0.05). This indicates increase in risk of death at longer pulse duration.

### Influence of species on fish survival with weight, length and condition factor held constant

In Table 3, positive parameter estimate indicates that Nile tilapia had lower risk of death (higher survival) than catfish exposed to atrazine, mancozeb and lambda cyhalothrin, while negative parameter estimate indicates that Nile tilapia had higher risk of death (lower survival) than catfish pulse-exposed - to chlorpyrifos (*p* < 0.05). Catfish pulse-exposed to atrazine, mancozeb, chlorpyrifos, and lambda cyhalothrin were 2.020, 1.875, 0.010, and 1.722 times more likely to die than Nile tilapia respectively. Thus risk of death for Nile tilapia exposed to atrazine, mancozeb and lambda cyhalothrin decreased by 102%, 87.5% and 72.2% compared to catfish respectively, while risk of death of catfish exposed to chlorpyrifos was 99% lower than Nile tilapia respectively.

**Table 3:** Cox PH model summary of species effect on survival of fingerlings.

Pesticides	df	Parameter estimate	SE	Chi-square	Pr > ChiSq	Hazard ratio
Atrazine*	1	0.703	.266	6.988	.008	2.020^a^
Mancozeb	1	0.628	.280	5.020	.025	1.875^a^
Chlorpyrifos*	1	− .4.643	.949	23.938	.000	.010^a^
Lambda cyhalothrin	1	0.543	.220	6.107	.013	1.722^a^

*Hazard ratio constant with time.

^a^Statistical significant (*p* < 0.05).

^b^Not significant (*p* > 0.05).

However in Table 4, the risk of death for Nile tilapia and catfish pulse-exposed to atrazine, mancozeb and lambda cyhalothrin differed significantly only in the group exposed for 60 minutes (*p* < 0.05). Risk of death for both species were similar in the groups exposed for 15, 30 and 45 minutes (*p* > 0.05). On the other hand, for each pulse length, risk of death from chlorpyrifos exposure differed significantly between Nile tilapia and catfish. Risk of death of 15, 30, 45 and 60 minutes pulse exposure to chlorpyrifos decreased by a factor of 104.94, 87.64, 117.72, and 83.52 respectively in catfish compared with tilapia fingerlings.

**Table 4:** Cox PH model summary of species effect on survival of fingerlings at different exposure duration.

Pesticide	Exposure duration (minutes)	Df	Parameter estimate	SE	Chi-square	Pr > ChiSq	Hazard ratio
Atrazine	15*	1	− .373	.646	.334	.563	.689^b^
	30*	1	− .539	.510	1.120	.290	.583^b^
	45*	1	− .373	.541	.476	.490	.689^b^
	60*	1	− 1.319	.486	7.364	.007	.268^a^
Mancozeb	15*	1	− .117	.671	.031	.861	.889^b^
	30*	1	− .498	.628	.629	.428	.608^b^
	45*	1	− .106	.557	.037	.848	.899^b^
	60*	1	− 1.514	.485	9.745	.002	.220^a^
Chlorpyrifos	15*	1	4.653	1.900	5.999	.014	104.944^a^
	30*	1	4.473	1.864	5.760	.016	87.638^a^
	45*	1	4.768	1.842	6.700	.010	117.715^a^
	60*	1	4.425	1.855	5.688	.017	83.515^a^
Lambda cyhalothrin	15*	1	.015	.519	.001	.977	1.015 ^b^
	30*	1	− .827	.440	3.524	.060	.437^b^
	45*	1	.208	.495	.177	.674	1.231^b^
	60	1	− 2.295	.739	9.636	.002	.101^a^

*Hazard ratio constant with time.

^a^Statistical significant (*p* < 0.05).

^b^Not significant (*p* > 0.05).

### Influence of pulse length on Survival of fingerlings with weight, length and condition factor held constant

Table 5 shows the mortality count and estimated median survival time for groups in which 50% mortality had occurred after pesticide pulse exposure.

**Table 5:** Mortality count and median survival time of fingerings pulse exposed to pesticides.

Fish species	Pesticide	Exposure duration	Total	Failed	Censored	Median survival time (h) 95% C.I		
							Lower	Upper
Catfish	Atrazine	15-m	20	6	14		60	
		30-m	20	11	9	77.5	33	
		45-m	20	8	12		50	
		60-m	20	15	5	53	27	96
		96-h*	20	17	3	9	5	60
	Mancozeb	15-m	20	5	15			
		30-m	20	7	13		92	
		45-m	20	7	13		75	
		60-m	20	16	4	7.5	2	51
		96-h*	20	20	0	9	8	15
	Chlorpyrifos	15-m	20	4	16			
		30-m	20	6	14		35	
		45-m	20	8	12		9	
		60-m	20	15	5	9	7	13
		96-h*	20	20	0	9	7	13
	Lambda cyhalothrin	15-m	20	7	13		3	
		30-m	20	13	7	2	2	
		45-m	20	7	13		1	
		60-m	20	20	0		1	
		96-h*	20	20	0		1	
Nile tilapia	Atrazine	15-m	20	4	16			
		30-m	20	6	14		11	
		45-m	20	6	14		70	
		60-m	20	6	14		75	
		96-h*	20	20	0	3	2	4
	Mancozeb	15-m	20	4	16			
		30-m	20	4	16			
		45-m	20	6	14		65	
		60-m	20	6	14		61	
		96-h*	20	20	0	6	5	8
	Chlorpyrifos	15-m	20	20	0	1.50	1	2
		30-m	20	20	0	2	1	2
		45-m	20	20	0	2	1	2
		60-m	20	20	0	2		
		96-h*	20	20	0	2		
	Lambda cyhalothrin	15-m	20	8	12		8	
		30-m	20	9	11		7	
		45-m	20	10	10		4	
		60-m	20	11	9	15.5	2	
		96-h*	20	20	0	2	1	2

C.I, Confidence interval. Median survival time and confidence interval was only estimated when 50% mortality occur; m-minutes, h-hours *- continuous exposure.

Pulse length was a fairly consistent predictor of pesticide pulse toxicity in the exposed fish. With other covariates held constant, there was a significant improvement (p< 0.05) in fit of the model with pulse length as predictor of survival for catfish fingerlings exposed to atrazine, mancozeb, chlorpyrifos, lambda cyhalothrin but not Nile tilapia (*p* > 0.05). In Table 6, hazard ratio < 1 and a significant negative parameter estimate indicates risk of death (pesticide hazard) for catfish exposed to atrazine, chlorpyrifos and lambda cyhalothrin decreased as pulse length decreased. The likelihood of catfish dying after 15 and 45 minutes pulse exposure compared to 60 minutes pulse exposure to atrazine decreased by a factor of 0.272 and 0.167 respectively (*p* < 0.05). Similarly, mancozeb hazard in catfish exposed for 15, 30 and 45 min relative to 60 min decreased by a factor of 0.143 (86%), 0.206 (79%) and 0.219 (78%) respectively (*p* < 0.05). Once more, the likelihood of death due to pulse exposure of 15, 30 and 45 minutes to chlorpyrifos decreased by a factor of 0.155 (85%), 0.242 (76%), and 0.368 (63%), respectively relative to 60 minutes, while lambda cyhalothrin hazard in catfish exposed for 15, 30 and 45 minutes decreased significantly by a factor of 0.136 (86%), 0.308 (69%) and 0.181 (82%) respectively compared with those exposed for 60 minutes.

**Table 6:** Cox PH model summary of effect of pulse duration on hazard of single pesticide.

Pesticide	Species		df	Parameter estimate	SE	Chi-square	Hazard ratio
Atrazine	Catfish*	15 min	1	− 1.300^a^	.484	7.221	.272^a^
		30 min	1	− .512^b^	.397	1.663	.599^b^
		45 min	1	− .928^a^	.438	4.483	.395^a^
		60 min	3			9.001	
	Tilapia	15 min	1	− .361^b^	.646	.313	.697^b^
		30 min	1	.141^b^	.578	.060	1.152^b^
		45 min	1	.051^b^	.577	.008	1.052^b^
		60 min	3			.654	
Mancozeb	Catfish	15 min	1	− 1.945^a^	.518	14.123	.143^a^
		30 min	1	− 1.577^a^	.459	11.826	.206^a^
		45 min	1	− 1.519^a^	.458	11.007	.219^a^
		60 min	3			23.341	
	Tilapia*	15 min	1	− .351^b^	.646	.296	.704^b^
		30 min	1	− .412^b^	.646	.407	.662^b^
		45 min	1	.037^b^	.577	.004	1.037^b^
		60 min	3			16.070	
Chlorpyrifos	Catfish*	15 min	1	− 1.864^a^	.567	10.818	.155^a^
		30 min	1	− 1.418^a^	.488	8.444	.242^a^
		45 min	1	− .999^a^	.441	5.130	.368^a^
		60 min	3			.779	
	Tilapia*	15 min	1	.246^b^	.317	.601	1.279^b^
		30 min	1	.045^b^	.316	.020	1.046^b^
		45 min	1	.836^a^	.334	6.249	2.307^a^
		60 min	3			7.694	
Lambda cyhalothrin	Catfish	15 min	1	− 1.994^a^	.499	15.958	.136^a^
		30 min	1	− 1.179^a^	.411	8.227	.308^a^
		45 min	1	− 1.710^a^	.489	12.202	.181^a^
		60 min	3			20.231	
	Tilapia*	15 min	1	− .547^b^	.465	1.385	.579^b^
		30 min	1	− .410^b^	.450	.832	.663^b^
		45 min	1	− .234^b^	.437	.286	.791^b^
		60 min	3			1.604	

*Hazard ratio constant with time.

^a^Statistical significant (*p* < 0.05).

^b^Not significant (*p* > 0.05).

On the other hand, atrazine, mancozeb and lambda cyhalothrin hazard were not significant different in Nile tilapia fingerlings exposed for 15, 30, and 45 minutes compared with those exposed for 60 minutes. Chlorpyrifos hazard in tilapia was also not significantly different in groups exposed for 15 and 30 minutes compared with those exposed for 60 minutes. Chlorpyrifos hazard however increased by a factor of 2.307 in fingerlings exposed for 45 minutes compared with those exposed for 60 minutes (*p* < 0.05) probably due to stochastic deaths.

### Combined influence of species, pulse length, weight, length and condition factor on survival of fingerlings

In Table 7, taking all predictors—species, pulse length, weight, length and condition factor into account, the probability of surviving atrazine pulse toxicity was about 9 times higher in tilapia compared with catfish. Hazard ratio < 1 and a significant negative parameter estimate indicates risk of death (hazard) of atrazine decreased as pulse length decreased and fish weight increased. The likelihood of death after 15, 30 and 45 minutes exposure decreased by a factor of 0.454, 0.309 and 0.457 respectively compared to 60 minutes (*p* < 0.05), while risk of death decreased by 100% in fishes weighing 0.1 gram more than another fish. Furthermore, significant positive parameter estimate and hazard ratio > 1 indicates increased risk of death for longer fingerlings and fishes with higher condition factor. The risk of death was 27.648 and 31.071 times higher in fishes longer than another by 1 cm and condition factor higher by 0.1. Longer fishes have more surface area which can facilitate uptake of pesticides during the pulse exposure than shorter fishes.

**Table 7:** Cox PH model summary of all predictors on survival of fingerlings.

Pesticide		Parameter estimate	SE	Chi-square	Df	Pr > ChiSq	Hazard ratio	95.0% CI for Hazard ratio	
								Lower	Upper
Atrazine	Species	2.197	.565	15.146	1	.000	9.002^a^	2.977	27.223
	15 min	− .789	.394	4.007	1	.045	.454	.210	.984
	30 min	− 1.175	.391	9.035	1	.003	.309	.144	.664
	45 min	− .784	.353	4.936	1	.026	.457	.229	.912
	60 min			11.018	3	.012			
	Weight	− 12.315	2.027	36.898	1	.000	.000	.000	.000
	Length	3.320	.706	22.128	1	.000	27.648	6.934	110.241
	Condition factor	3.436	.660	27.081	1	.000	31.071	8.517	113.351
Mancozeb	Species	.859	.541	2.520	1	.112	2.360^a^*	.817	6.816
	15 min	− 1.581	.416	14.464	1	.000	.206	.091	.465
	30 min	− 1.639	.395	17.235	1	.000	.194	.090	.421
	45 min	− 1.474	.368	16.017	1	.000	.229	.111	.471
	60 min			27.527	3	.000			
	Weight	− 2.856	1.918	2.216	1	.137	.058	.001	2.469
	Length	− 2.223	.876	6.433	1	.011	.108	.019	.604
	Condition factor	− .697	.850	.673	1	.412	.498	.094	2.634
Chlorpyrifos	Species	− 12.725	51.934	.060	1	.806	.000^a^*	.000	4.8E + 38
	15 min	− .428	.290	2.180	1	.140	.652	.369	1.151
	30 min	− .406	.270	2.266	1	.132	.666	.393	1.130
	45 min	.216	.271	.634	1	.426	1.241	.729	2.113
	60 min			6.404	3	.094			
	Weight	.107	.525	.042	1	.838	1.113	.398	3.116
	Length	.061	.327	.035	1	.852	1.063	.560	2.016
	Condition factor	.066	.220	.089	1	.765	1.068	.694	1.642
Lambda cyhalothrin	Species	3.646	.574	40.291	1	.000	38.32^a^	12.431	118.125
	15 min	− 1.375	.356	14.925	1	.000	.253	.126	.508
	30 min	− .652	.321	4.124	1	.042	.521	.278	.978
	45 min	− .830	.328	6.412	1	.011	.436	.229	.829
	60 min			15.783	3	.001			
	Weight	− 2.033	.863	5.543	1	.019	.131	.024	.711
	Length	− .652	.364	3.216	1	.073	.521	.255	1.063
	Condition factor	.342	.281	1.486	1	.223	1.408	.812	2.442

^a^Increase in hazard ratio, while b indicates decrease in hazard ratio compared with when other predictors where held constant.

*Significant when other predators were held constant but no longer significant.

^+^Not significant when other predators were held constant and not significant now.

^#^Not significant when other predators were held constant but now significant.

After accounting for species, pulse length, weight, length and condition factor, mancozeb pulse toxicity was only significantly associated with pulse length and fish length. Hazard ratio < 1 and a significant negative parameter estimate indicates the likelihood of death after 15, 30 and 45 minutes exposure to mancozeb decreased by a factor of 0.206, 0.194 and 0.229 respectively compared to 60 minutes (*p* < 0.05), while the risk of death decreased by 89.2% in fishes longer by 1 cm.

Chlorpyrifos pulse toxicity was associated with species alone. The probability of surviving chlorpyrifos toxicity decreased by 100% in catfish compared to Nile tilapia.

Lambda cyhalothrin was associated with species, pulse length, and fish weight. The probability of survival was 38.32 times higher in tilapia compared with catfish. Hazard ratio < 1 and a significant negative parameter estimate indicates the likelihood of death after 15, 30 and 45 minutes exposure to lambda cyhalothrin decreased by a factor of 0.253, 0.521 and 0.436 respectively compared to 60 minutes exposure (*p* < 0.05).Risk of death decreased by 86.9% in fishes weighing 0.1 gram more than another fish.

## Discussion

The pesticide concentrations used in this study may be considerably higher than background concentrations in the environment. The use of high concentration in pulse assessment enables effects to be adequately characterized and provides a basis for estimating impacts at the predicted environmental concentration^31^. Moreover, in aquatic ecosystems adjacent to agricultural lands, peak pesticide concentration may be reached during rainfall events shortly after field application and could be at least 20-fold higher than the background concentrations^22^.

As a herbicide, atrazine causes the reversible inhibition of photosynthesis in photosystem II in plants. However in fish and other animals, it might be genotoxic, clastogenic, and affect hormones, and biochemical processes^33^. Its solubility in water is about 30 mg/L at 20 ^o^C^6^ and also readily metabolized in fish, excreted and does not bio-concentrate in tissues^34^. On the other hand, mancozeb (manganese–zinc ethylenebis dithiocarbamate) inhibits enzyme activity in fungi by forming a complex with metal-containing enzymes including those that are involved in the production of ATP. Its chelating properties possibly interferes with a number of enzyme systems that contain metals, such as zinc, copper, and iron (e.g., dopamine b-hydro xylase)^35^. Its solubility in water is about 6.4 mg/L at 25°C^36^. Lambda cyhalothrin, blocks voltage-gated sodium channels present on neuronal axons in brain and muscles^37^ causing swift paralysis and death to insects. It has solubility of 5 × 10^−3^ mg/L in purified water^13^. Chlorpyrifos has low water solubility and can bio-concentrate in the liver, intestine and gills^38^. It has a specific mode of action which involves preventing the breakdown of acetylcholine by inhibiting acetylcholinesterase activity. The resulting accumulation of acetylcholine in the synaptic cleft causes overstimulation of the neuronal cells, which leads to neurotoxicity and eventually death^39^.

Catfish was expected to have higher survival time (lower risk of death) than Nile tilapia given that catfish is considered sturdy and more tolerant to stressors than Nile tilapia^40–42^ . Also, higher LC_50_ values for atrazine, chlorpyrifos and lambda cyhalothrin were previously reported for catfish compared with tilapia^43–47^ indicating tilapia may be more sensitive to pollutants. However in this study, the trend of the risk of death of both species was not consistent with our assumptions after pulse exposure to pesticides. Nile tilapia had significantly lower risk of death than catfish after pulse exposure to atrazine, mancozeb, and lambda cyhalothrin particularly after 60 minutes. Catfish however had a lower risk of death than tilapia after exposure to chlorpyrifos irrespective of pulse length. This differential response is consistent with a previous study^31^ which reported that *Calineuria californica* was less sensitive to carbaryl than *Cinygma sp.* only after pulsed exposures but similar after 96 hours of continuous exposure. It is not clear why tilapia had lower risk of death than catfish after 60 minures pulse-exposure to atrazine, mancozeb, and lambda cyhalothrin. Pesticide physicochemical properties, differences in uptake rate and time to equilibration, morphological differences, as well as faster recovery due to differential pesticide metabolism could probably explain the differences in the two species detected in this study.

The similarity in the risk to death of fingerlings exposed to atrazine, mancozeb and lambda cyhalothrin for 15, 30, and 45 minutes suggest that the difference between the two fish species after 60 minutes pulse-exposure probably lie in quicker toxico-dynamic recovery suggesting a more efficient biotransformation of pesticide in Nile tilapia. Previous authors have shown that phase I and II biotransformation efficiency varies among finfish with some categorized as “more efficient metabolizers” than others^48^ . Mancozeb and lambda cyhalothrin could bio concentrate in tissues given their low water solubility. Moreover, the gill filaments of catfish are thicker than Nile tilapia and catfish have small opercula opening in comparison to tilapia species which enables the fish to trap moisture between the filaments^49^ thus facilitating the uptake of pesticides by the gills. Also, there are more mucous cells in the filamentary epithelium and lamellae of catfish than tilapia indicting a high mucous secreting character of the catfish gills and more ability to trap pesticides in the oral cavity^49^. Differences in the morphology of the cerebellum of catfish and tilapia could explain the high risk of death in tilapia. The cerebellum is the main organ responsible for balance and equilibrium of the body and coordination and a possible target for chlopyrifos. While the size of catfish cerebellum is about four times greater than that of tilapia of same body weight, it consists of three strata- an outer molecular layer, intermediate layer of purkinje cells and a thick deeper layer of granular cells. In contrast, the granular layer is ill defined in the tilapia species while the granule cells are widely distributed^50^. Catfish may experience delayed chlorpyrifos toxicity due to the bigger cerebellum size and clearly defined strata.

The effect of pulse length rather than pulse concentration was the focus in this study. Earlier study^51^ showed that pulse exposure to pesticides may cause more hazard than continuous exposure. This is consistent with the results of this study in which the risk of death after 60 minutes pulse exposure was similar to continuous exposure for 96 hour. Time is an important variable in toxic response. Critical threshold dose resulting in adverse effect could occur after a “short” time^52^. Data from this study suggests 60 minutes pulse may be a critical exposure time for pulse exposure to high pesticide concentration below which toxicity is minimal. Previous study provides support for this. For instance, 50% mortality was only reached for *C. californica* exposed to 1,730 mg/L carbaryl for 60 minutes but not after 15 or 30 minutes pulse exposure^31^. Survival time presents a way to express individual/species tolerance of toxicants. Survival time after pulse exposure reflects “individual tolerance” as it represent the time for which biological processes like, absorption, distribution, bioaccumulation, and onset of cellular/physiological impairment have occurred leading to either death or recovery for each fingerlings.

Fish weight, length and condition factor are intrinsic variables that may influence the survival of pulsed-exposed fishes. Risk of death in fingerlings exposed to pesticide was anticipated to decrease as fish weight increased. In line with our expectations, risk of death decreased as weight of fingerings increased particularly in the groups exposed to atrazine and lambda cyhalothrin given the slight differences in the weight of fishes exposed to different pulse lengths of each pesticides. Chlorpyrifos and mancozeb pulse toxicity were not associated with fish weight due to similar fish weights exposed to each pesticide for different duration. Also, results from this study suggests risk of death of pulse exposure to pesticides may decrease as length and condition factor of fingerings increase. The condition factor (CF) of a fish is often used to depict the health condition of a fish^53^, so the higher the condition factor, the healthier or fit the fish. Risk of death from pulse exposure to atrazine decreased as fish length and condition factor increased. Length of fish species exposed to atrazine differed slightly. Survival probability may have increased as weight, length and condition factor increased because bigger and fitter fingerlings may have higher tolerance for stress than smaller fingerlings. This is consistent with previous study^29^ where the sensitivity of fishes exposed to benzocaine where higher for smaller fishes than bigger fishes. Decrease in risk of death with increasing size is consistent with individual tolerance concept^54^.

## Conclusion

In this study, intrinsic (specie difference, weight, length and condition factor) and extrinsic factors (pulse length) influenced risk of death of fingerlings exposed to pesticides. Generally, the hazard of pesticide pulse exposure decreased as fingerling size (weight, length and condition factor) increased but increased as pulse length increased. Nile tilapia fingerlings were  more susceptible to continuous pesticides exposure than catfish, but appeared to be less susceptible to pulse exposure. Brief exposure of fingerlings to pesticides 60 minutes was as hazardous as continuous exposure for 96 hours to some pesticides. Data from this study suggests 60 minutes pulse may be a critical exposure time for pulse exposure to high pesticide concentration.

## Data Availability

All data generated or analysed during this study are included in this published article and its supplementary information files.
